# Coral reef biofilm bacterial diversity and successional trajectories are structured by reef benthic organisms and shift under chronic nutrient enrichment

**DOI:** 10.1038/s41522-021-00252-1

**Published:** 2021-12-01

**Authors:** Kristina L. Remple, Nyssa J. Silbiger, Zachary A. Quinlan, Michael D. Fox, Linda Wegley Kelly, Megan J. Donahue, Craig E. Nelson

**Affiliations:** 1 Daniel K. Inouye Center for Microbial Oceanography: Research and Education, Department of Oceanography and Sea Grant College Program, University of Hawaiʻi at Mānoa, Honolulu, HI USA; 2 Department of Biology, California State University, Northridge, CA USA; 3 Department of Biology, San Diego State University, San Diego, CA USA; 4 Scripps Institution of Oceanography, University of California, San Diego, CA USA; 5 Woods Hole Oceanographic Institution, Woods Hole, MA USA; 6 Hawaiʻi Institute of Marine Biology, University of Hawaiʻi at Mānoa, Honolulu, HI USA

**Keywords:** Biofilms, Microbial ecology, Water microbiology

## Abstract

Work on marine biofilms has primarily focused on host-associated habitats for their roles in larval recruitment and disease dynamics; little is known about the factors regulating the composition of reef environmental biofilms. To contrast the roles of succession, benthic communities and nutrients in structuring marine biofilms, we surveyed bacteria communities in biofilms through a six-week succession in aquaria containing macroalgae, coral, or reef sand factorially crossed with three levels of continuous nutrient enrichment. Our findings demonstrate how biofilm successional trajectories diverge from temporal dynamics of the bacterioplankton and how biofilms are structured by the surrounding benthic organisms and nutrient enrichment. We identify a suite of biofilm-associated bacteria linked with the orthogonal influences of corals, algae and nutrients and distinct from the overlying water. Our results provide a comprehensive characterization of marine biofilm successional dynamics and contextualize the impact of widespread changes in reef community composition and nutrient pollution on biofilm community structure.

## Introduction

Biofilms are complex communities of surface attached microorganisms that are encased in an extracellular polymeric matrix^1^. They are ubiquitous in aquatic environments where they provide ecosystem services including primary production^2^, organic matter decomposition^3^, and nutrient cycling^4^. The close proximity of cells and the ability of the extracellular matrix to capture and retain nutrients from the surrounding fluid make biofilms “hot spots” of biogeochemical cycling^5^. Extrinsic factors including fluid dynamics and nutrient availability have been shown to influence biofilm community composition, cell density, and rate of succession^6–9^. In marine environments biofilms are known to play key roles in the settling and subsequent metamorphosis of invertebrate larvae^10,11^, an ecosystem service that is likely governed by the community composition of the biofilm^10,12^.

Although the importance of marine biofilms is widely accepted, most coral reef microbiology studies have focused on planktonic and symbiotic organisms. Studies focusing on bacterioplankon communities on coral reefs have revealed active and dynamic heterotrophic bacterial assemblages influenced by reef residence time^13–15^, diel ecosystem processes^16^, sources and concentrations of organic matter^13,17,18^, reef benthic composition^19–22^, direct coral interactions^23,24^, and nutrient availability^20,21^. Similarly, populations of potentially pathogenic bacteria and virulence genes increase in the surface mucus layer of corals in response to increased nutrient loads^25,26^; and the microbiomes of physiologically sensitive corals may experience an overall decrease in microbial diversity accompanied by an increase in disease-associated microbes with ocean acidification and warming^27^. In combination with other environmental stressors, nutrient pollution in coastal ecosystems is implicated in shifts away from coral dominated reefs toward those dominated by fleshy algae^28,29^. These changes in benthic cover may further impact the physical and chemical environment by altering physiological responses of the benthic community such as coral and algal photosynthesis^30,31^, coral and algal organic matter exudation^17^ and ultimately impacting net community production and calcification rates^32^. Organic matter exudates produced by algae have been shown to be compositionally distinct from those of corals^17,33^, potentially driving the restructuring of the planktonic microbial community as reefs shift to algal dominance^19,20,33–36^, yet it is unclear how shifts in organic matter may affect biofilm microbial communities.

Because marine biofilms are important in mediating reef processes and maintaining biodiversity (e.g., nutrient cycling, larval settlement, etc.)^17,37,38^, a comprehensive understanding of the taxonomic structure and function of marine biofilms is crucial for adequately assessing the ecosystem services they provide and evaluating their role in maintaining coral reefs. Further, the use of biofilms in the assessment of pollution and ecosystem recovery reveals shifts in microbial community structure and function in response to environmental perturbation^39^. In this way, biofilms can serve as indicators of ecosystem health. Understanding the environmental factors that determine biofilm community assembly and succession are the first steps toward a predictive characterization of biofilm structure and function. As biofilms are sourced from and subject to the surrounding water column^40^ physical and chemical changes in the overlying water, as well as alterations in the planktonic microbial community, could significantly impact marine biofilm communities. Succession in marine biofilms has been previously studied^41–43^ finding that surface associated microbial communities differentiate from planktonic communities within hours^1,41,44^ and quickly progress from communities of primary settlers to established heterotrophic communities within a matter of days^45^. The very first stages of biofilm establishment are governed by the population of microorganisms capable of attaching to a surface^1,45^, which can be affected by physio-chemical properties of the substrate including rugosity, hydrophobicity, and the nutrient content of the organic matter coating the surface (i.e., the conditioning film) of the substrate^1^.

**Fig. 1:**
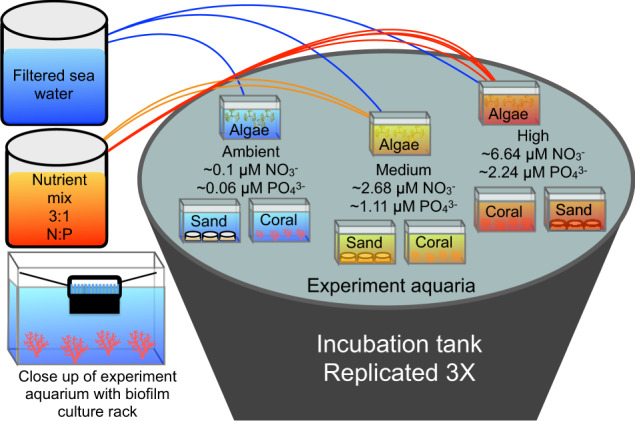
Experimental design. Three 1300 L flow-through incubation tanks were used to maintain constant temperature for nine flow-through aquaria each (total 27 experimental aquaria, 6 L volume each); a schematic of one tank with nine aquaria is shown here. Each aquarium held one of three benthic constituent organisms (algae, coral, or sand) and was supplied via peristaltic pump with filtered seawater amended to one of three nutrient treatments (ambient, low, or high) from independent header mixing aquaria (one for each nutrient level in each tank to maintain independence). Biofilms were cultured on glass slides, evenly spaced in slide racks and suspended vertically in each experimental aquarium to allow water to flow around each surface of the slide. Nutrients were measured weekly and were stable throughout the experiment; averages are reported. Additional data in Quinlan et al.^17^ and Silbiger et al.^32^.

Presented here are the results of an experiment designed to test the core hypotheses that the organic resources derived from the surrounding benthic organisms and inorganic nutrient availability work interactively and orthogonally to influence biofilm assembly and succession. Over the course of six weeks, we factorially tested the effect of constituent benthic organisms (corals, algae, and sand-associated microphytobenthos) and sustained nutrient loads (continuous micromolar enrichments in nitrate and phosphate at three concentrations) on the successional dynamics of developing marine biofilms on glass slides (Fig. 1). We hypothesized that biofilm communities would differ from planktonic communities, that biofilms would differ between benthic organismal treatments, and that nutrient amendments would further differentiate microbial communities.

## Results

### Multivariate analysis of bacterial community structure variation

**Fig. 2:**
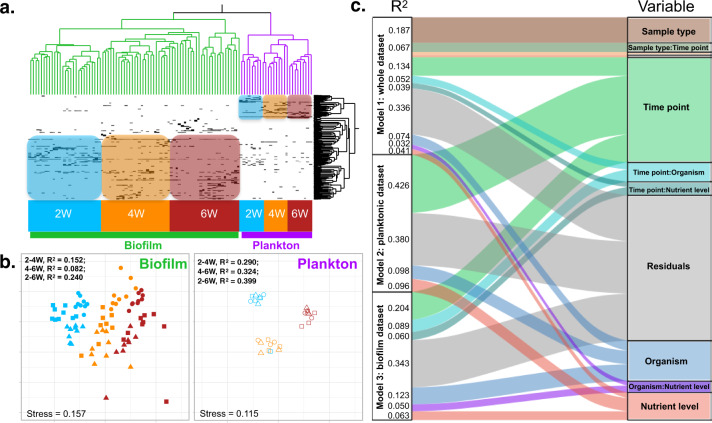
Multivariate visualization and statistical differentiation of biofilm and planktonic bacterial communities. Hierarchical clustering (Ward’s minimum variance method) organized all OTUs in the study according to distributions of standardized (*z*-scored) mean relative abundance across treatments. Throughout the 6-week experiment, biofilm communities were distinct from planktonic microbial communities while both clustered by time point (**a**). Biofilms displayed a successional trajectory not found in the planktonic community (**b**); data points are color coded by time point (2-weeks = blue, 4-weeks = gold, 6-weeks = red), and shaped by sample type and organism (open = planktonic, closed = biofilm; Triangle = algae, circles = coral, square = sand). Variance partitioning of three different PERMANOVA models (**c**) illustrate the relative influence of sample type, time point, benthic organism, and nutrient enrichment on bacterial taxonomic structure of planktonic and biofilm communities. Model 1 combines both planktonic and biofilm communities and emphasizes that sample type is the strongest driver of microbial community composition. Time point has the next largest influence on both planktonic (Model 2) and biofilm (Model 3) bacterial communities. Biofilm microbial communities are more strongly influenced by benthic organism (*R*^2^ = 0.123) than nutrient treatment (*R*^2^ = 0.063), while these parameters are equally influential in the planktonic community, explaining a much smaller variance (<1%). All tests and model terms shown are significant (*p* < 0.01).

Planktonic and biofilm communities differed clearly across all time points and both clustered primarily by time point (Fig. 2a). Specifically, a strong differentiation was observed between biofilm communities and planktonic communities across all time points (sample type *R*^2^ = 0.187, *p* = 0.001), and each time point differed significantly, independent of sample type (time point *R*^2^ = 0.134, *p* = 0.001; Fig. 2c, Model 1 and Table S3). Mean bacterial diversity differed significantly between sample types (*p* < 0.01), with evenness consistently higher in biofilms (mean = 0.879 ± 0.0479 sd) than in bacterioplankton communities (mean = 0.731 ± 0.0783 sd) at each time point (*p* < 0.001) and richness higher in biofilms by 6 weeks (*p* = 0.002) (mean richness at 6 weeks biofilm = 215.292 ± 82.95 sd, bacterioplankton = 149.596 ± 32.65 sd). Additionally, temporal variation in microbial communities differed between biofilms and plankton (Fig. 2c, Model 1: sample type × time point *R*^2^ = 0.067, *p* = 0.001), indicating that successional patterns observed in the biofilms were orthogonal to changes observed in the planktonic community.

Because of the clear distinction between planktonic and biofilm communities (Fig. 2a), we additionally evaluated the fixed single and 2-way interactive effects of time, benthic organism, and nutrient treatments on biofilm and planktonic communities separately (Fig. 2b, c and Supplementary Table 2). Since planktonic samples were pooled across replicates and primarily used as a reference for biofilm responses, interaction terms could not be evaluated in the plankton community. Within each sample type (biofilm or bacterioplankton), microbial community structure changed significantly through time (Fig. 2b: Models 2 and 3, *p*_time_ < 0.001) and more than twice of the explained variance was attributed to time point in the planktonic community than in the biofilm community (Fig. 2b: Models 2 and 3; *R*^2^ = 0.426 and 0.204, respectively). Relative to the strong directional succession of the biofilm community the planktonic community did not exhibit a clear directional trajectory, instead changing stochastically through time (Fig. 2b). Pairwise PERMANOVA emphasized that successional patterns observed in the biofilm community follow a directional trajectory with greater changes observed between 2 and 6 weeks (*R*^2^ = 0.240, *p* < 0.001) than between 2 and 4 or 4 and 6 weeks (*R*^2^ = 0.152 and 0.082, respectively, *p* < 0.001) while planktonic communities were roughly equidistant between 2, 4, and 6 weeks (*R*^2^ = 0.290–0.399, each *p* < 0.001; Fig. 2b). We hypothesized that the composition of the benthic reef community (henceforth “organism” would influence biofilm communities from early successional stages. We further hypothesized that increasing inorganic nutrients could alter biofilm communities either indirectly, such as by influencing exudates of benthic organisms^17^, or directly by shifting nutrient dependence away from autochthonous (within biofilm, presumably organic) sources toward environmentally available inorganic sources. Both organism and nutrient manipulations had significant effects on biofilm community structure (*p* < 0.001) across all time points. Moreover, significant two-way interactions were identified among all three variables (*p* < 0.01); a 3rd degree factorial model was also tested in biofilm communities but no significant three-way interaction (*p* = 0.296) was identified. The variance explained by the temporal effect (*R*^2^ = 0.204, Fig. 2c: Model 3) was greater than that of the organism (*R*^2^ = 0.123) or nutrient effect (*R*^2^ = 0.063), demonstrating that successional changes over time were the primary overall driver of community differentiation in these biofilms (Fig. 2b, c).

### Comparison of biofilm communities between time points

**Fig. 3:**
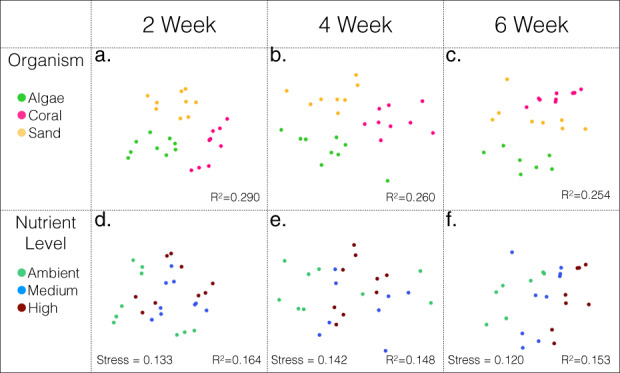
Effects of benthic organism and nutrient enrichment on biofilm communities within each time point. At each time point, both nutrient treatment and benthic organism significantly influence biofilm community structure (*p* < 0.001). Organism (**a**–**c**) is a better predictor of biofilm community than nutrient level (**d**–**f**) across each time point. A significant interaction between organism and nutrient level is also observed at each time point: 2 week (*R*^2^ = 0.140, *p* = 0.003), 4 week (*R*^2^ = 0.144, *p* = 0.031) and 6 week (*R*^2^ = 0.156 & *p* = 0.006), indicating that nutrients affected community structure differentially according to the benthic organism present.

Because biofilm community structure differed most strongly by time point (Fig. 2), to better clarify how marine biofilms are influenced by benthic organisms and inorganic nutrients we separately analyzed the biofilm community differentiation by organism and nutrient treatments within each time point (Fig. 3). Both organism and nutrient level were found to significantly impact the resulting biofilm communities at each time point, with organism consistently explaining more variance between biofilm communities than nutrient level (~26% compared with 15%) (Fig. 2). Organism and nutrient effects were increasingly orthogonal through time and significant interactions between organism and nutrients strengthened over time (2-week *R*^2^ = 0.140, *p* = 0.003; 4-week *R*^2^ = 0.144, *p* = 0.031; 6-week *R*^2^ = 0.156, *p* = 0.006), suggesting that the chronic nutrient amendments integrated their effects across successional processes. To estimate the variability in community composition of a given treatment, multivariate dispersion was measured among time points as well as within each time point grouped by organism or nutrient level (Supplementary Fig. 2). The average distance to centroid (dispersion) increased between 2-week and 4-week time points (*p* = 0.004), but dispersion did not change from 4 to 6 weeks (*p* = 0.993). Dispersion was significantly different between organismal treatments only at 6-weeks (*p* = 0.048), where coral treatments displayed a significantly lower average distance to centroid than sand and algae treatments. Biofilms that were subject to a medium nutrient addition displayed significantly lower dispersion at 2-weeks compared with ambient and high-nutrient treatments (*p* = 0.0148), but there were no significant differences among nutrient treatments at later time points.

**Fig. 4:**
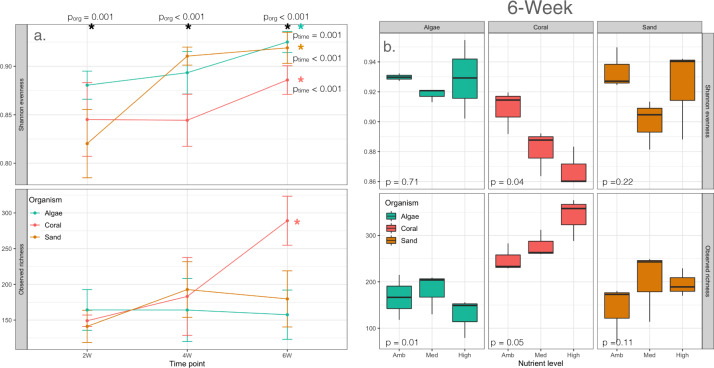
Changes in alpha diversity metrics of bacteria richness and evenness in biofilms through time in response to benthic organism and nutrient treatments. **a** Richness and evenness significantly increased over 6 weeks (*p*_time_ < 0.001) independent of the benthic organism with which they were cultured (*p*_org_ < 0.001). Black asterisks indicate time points with significant organismal effect (*p*_org_ ≤ 0.001). Color coded asterisks indicate an increase in richness or evenness between 2 and 6 weeks (*p*_time_ ≤ 0.001; green = algae, pink = coral, yellow = sand). **b** Within each organismal treatment, nutrients differentially impact biofilms at 6 weeks and had opposing effects on richness and evenness in coral treatments.

Univariate linear mixed effect models were used to test hypotheses regarding the effects of time and treatments on alpha diversity estimates (Observed Richness and Shannon Evenness) within biofilm communities (Fig. 4). Both richness and evenness of biofilm bacterial communities differed significantly among time points, between benthic organisms and their interaction (*p* < 0.001). Evenness additionally responded to nutrients (*p* = 0.004) and this effect differed among organismal treatments (*p* = 0.016). Overall, biofilm bacterial community evenness increased with succession (2-week mean = 0.849 ± 0.0514 sd; 6-week mean = 0.909 ± 0.0268 sd), with significant differences between 2 and 6 weeks within every organismal treatment (pairwise Tukey *p* < 0.05). Richness only significantly increased in biofilms cultured with corals (Coral *p* < 0.001; Fig. 4a). At each time point, an organismal effect on evenness was observed (2-week *p* = 0.012; 4-week and 6-week *p* < 0.001; Fig. 4a). However, a significant organism effect on richness was only observed at 6 weeks (Fig. 4a), indicating that organismal impacts on biofilm diversity took time to manifest. Nutrients exhibited effects on evenness at 2 weeks and 6 weeks (2 week *p* = 0.03; 4 week *p* = 0.4; 6 week *p* = 0.004) but nutrients only effected richness at 6 weeks (2 weeks = 0.1; 4 weeks *p* = 0.3; 6 weeks *p* = 0.04); a significant organism-nutrient effect on richness was observed at both 2 and 6 weeks (*p* < 0.01) but this interaction did not manifest in evenness (*p* = 0.8), indicating that nutrients again played a subtle role in diversity effects.

### Population level analyses

Most bacterial taxa differed at least qualitatively in relative abundance between biofilm and planktonic communities throughout the experiment (Fig. 2a). Twelve families significantly differed between the two sample types and individually comprised more than 2% of the total reads in either community (Fig. S5). Surface attached microbial communities were enriched with Rhodobacteraceae, Rhizobiaceae, and Hyphomonadaceae of the Alphaproteobacteria, making up ~19%, 5%, and 4% of the biofilm total reads, respectively. Further, Parcubacteria (3%), Nostocales (4%), Microtrichaceae (2%), Saprospiraceae (5.4%) and Pirellulaceae (3.5%) were also significantly enriched in biofilm communities. The free-living community was enriched with SAR11 (8.9%), Vibrionaceae (6.2%) and the Bacteroidetes families Flavobacteriaceae (12.5%) and Cryomorphaceae (16.1%).

**Fig. 5:**
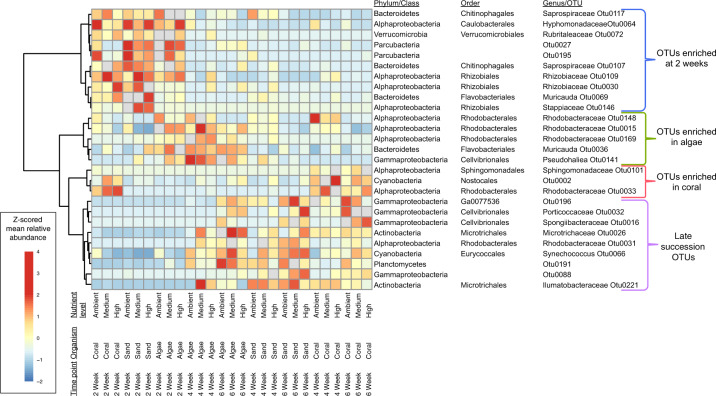
Visualization of enrichment of biofilm OTUs associated with treatments and time points. Hierarchical clustering (Ward’s minimum variance method) organized statistically selected OTUs according to distributions of standardized (z-scored) mean relative abundance across treatments. OTUs that were both significant by linear mixed model and discriminant by random forest are displayed along the *y*-axis. Note the stronger similarity between selected OTU enrichment patterns at earlier stages of development compared with later time points. Later time points separate primarily according to benthic organism, and further cluster by time point within these groups.

To identify bacterial taxa most strongly associated with time and treatment effects in the biofilm community level analyses, we used linear mixed models and a random forest algorithm to identify and rank OTUs that changed significantly. To ensure that our method was not selecting rare taxa, we additionally excluded OTUs with a mean relative abundance <0.05%. In all, 28 OTUs were selected by our criteria as associating significantly with experimental treatments of successional stage and benthic organism. Of these, the relative abundance of 12 OTUs responded significantly (adj. *p* ≤ 0.05) only to time; three OTUs responded only to organismal treatments; seven OTUs responded significantly to organism, time, and the time–organism interaction, and one OTU responded to both time and organism treatments, but did not exhibit a response to their interaction. These treatment associated OTUs indicate that marine biofilms from early time points were more similar to each other than at later time points which diverged according to the benthic organism with which the biofilms were cultured (Fig. 5). Early time points were marked by members of Parcubacteria, as well as the Saprospiraceae and Rhizobiaceae families, which were nearly absent from later time points (Fig. 5). Trends could be identified at higher taxonomic levels in later time points, which were enriched with constituents of the Planctomycetes, Actinobacteria, and Acidobacteria phyla (Fig. 4 and Supplementary Fig. 4). Gammaproteobacteria grew more abundant with time in all of the treatments (Supplementary Fig. 4) and were particularly important indicators of biofilms cultured with sand and algae treatments. In these treatments, OTUs classified as Porticoccaceae and Halieaceae of the gammaproteobacterial Cellvibrionales order were enriched in both algae and sand treatments at 4 and 6 weeks (Fig. 5 and Supplementary Fig. 5). Biofilms cultured with algae were additionally marked by multiple OTUs identified as members of the Rhodobacteracea family (Fig. 5). Cyanobacteria, especially members of the Nostocales order, were indicative of coral treatments throughout, while members of the Sphingomonadaceae family and the genus Synechococcus were indicative of coral and sand treatments at 4 and 6 weeks (Fig. 5 and Supplementary Fig. 5).

**Fig. 6:**
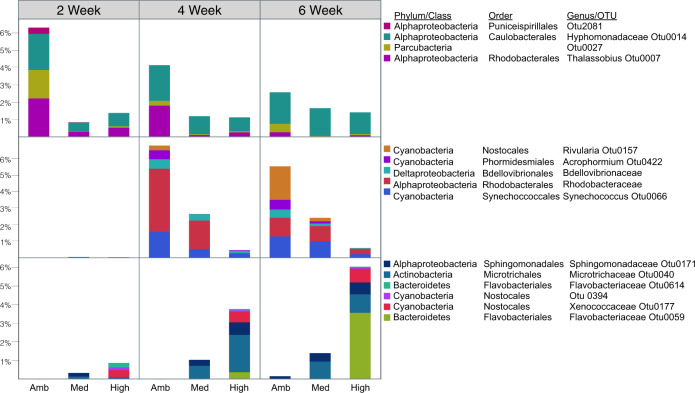
Relative abundance patterns of coral-associated biofilm OTUs that responded to nutrients. At top are families with OTUs that associated with earlier time points and decreased with nutrient additions, in the middle are families with OTUs that are associated with later time points that decrease with nutrient additions, and at bottom are families with OTUs associated with later time points that increase with nutrient additions.

The nutrient effect on biofilm communities was relatively small compared with the effects of succession and benthic organism (Fig. 2c), and, at the population-level, we found no OTUs consistently responding to nutrient treatments across all organismal treatments at any given time point. However, a significant nutrient–organism interaction was observed in 6-week coral treatments (Fig. 4). Thus, to further investigate the influence of nutrient amendments within our biofilms, we designed an algorithm to select OTUs associated with coral treatments and used linear models to identify OTUs that significantly responded to nutrient additions. In general, these coral associated OTUs were statistically associated with either early or late time points, and nutrient amended vs. unamended (ambient) treatments. Of the 23 OTUs that met our criteria, 13 were enriched at late time points (five from ambient, eight nutrient amended) and six OTUs were indicative of early time points (three ambient, three nutrient amended) (Fig. 6). Members of the Bdellovibrionales and Phormidesmiaceae are abundant at later time points but tend to decrease with nutrients, while OTUs of the Sphingomonadaceae increase with nutrient additions at later time points. Similarly, Puniceispirillales decreased with the addition of nutrients, but were enriched in early time points.

## Discussion

This study comprises the first thorough characterization of colonization and differentiation of Bacteria between the plankton and surface attached lifestyles in coral reefs (Supplementary Fig. 4). We demonstrate that biofilms exhibit a successional trajectory that diverges from the stochastic temporal dynamics of the bacterioplankton (Fig. 2), and use this successional trajectory to contextualize the degree to which benthic organismal context and nutrient enrichment influence biofilms (Fig. 3). Successional dynamics drive much of the variation among biofilms, with benthic context the next dominant driver and nutrient enrichment having the smallest effect (Fig. 2c). Metrics of diversity, taxonomic richness, and evenness change through time and with treatment, demonstrating that taxonomic evenness increases as marine biofilms mature, and that both benthic community member and nutrient availability affect biofilm community richness (Fig. 4). Our study demonstrates that organic resources from the surrounding benthic community and inorganic nutrient availability independently influence the assembly and succession of marine biofilm communities, and we provide a synthetic overview of the bacterial clades that differentiate coral and algal biofilms and respond to chronic nutrient enrichment (Figs. 5 and 6).

Microbial communities are strongly influenced by their physical and chemical surroundings. In marine environments, two major lifestyles are commonly observed: free-living bacterioplankton and surface-associated biofilm communities. It is argued that most marine microbes appear to prefer one lifestyle over the other, though preference may depend on environmental conditions^5^. For example, while surface attached communities can be heavily impacted by flow regimes^46^, bacterioplankton may be more influenced by residence time^13^ or diel processes^16^. Planktonic communities of Bacteria in the ocean are reportedly abundant in Pelagibacterales (SAR11), Puniceispirillales (SAR116), Flavobacteriales, Rhodospirilalles, the Gammaproteobacteria SAR86 clade, Synechococcales, and Actinomarinales^47–49^ while surface-attached communities are commonly enriched with Rhodobacterales, Alteromonadales, and specific groups of Cyanobacteria^48–50^. Nutrient input and availability is known to shift both surface attached and planktonic microbial communities. Although bacterioplankton communities in coastal areas can be relatively similar to those found in the open ocean^51^, coastal sites differ markedly in terms of nutrient input and production, and subtle shifts in the bacterioplankton community have been consistently observed in coral reefs relative to the adjacent ocean^13,52,53^. Betaproteobacteria appear to be confined to coastal regions^53,54^ and the predominant group of Cyanobacteria may be shifted from Prochlorococcus to Synechococcus in nutrient rich conditions like those found around coral reefs^13,55,56^. Our results support previous studies and find high abundances of typical bacterioplankton groups in the water. Because our experiment allowed for filtered seawater to flow through experiment tanks, variability in the bacterioplankton community of the source water likely contributed to the variability observed in our experiment tanks. In comparison biofilm communities were more evenly distributed, at least in part due to their ability to adhere to surfaces and were enriched with taxa that are known to benefit from surface attached lifestyles. Members of the Marine Roseobacter Clade (e.g., Rhodobacteraceae) are known copiotrophs for which surface colonization allows for more rapid response to labile organic matter^5^ and Planctomycetes (e.g., Pirellulaceae) have been found to dominate marine biofilms associated with kelp and are notable for their ability to degrade sulfate polymeric carbon compounds^57^. As well as taxa that are likely to benefit from the close proximity of cells found in biofilms such as Parcubacteria, which are known for their unusually small genomes and are thought to participate in symbiotic or episymbiotic interactions^58^ (Supplementary Fig. 3).

To date, studies investigating surface-attached or particle-attached microbial communities are typically conducted on found particles (i.e., marine snow or plastic debris) and, in reef ecosystems, focus on epiphytic-biofilms or host-associated microbiomes. Relatively few studies compare the successional stages of marine biofilms, and those that do tend to focus on the short time periods of primary colonizers and the initial biofilm formation that occurs on time frames of hours to days. This has left a knowledge gap regarding biofilm development and successional dynamics in marine systems and the environmental factors influencing any observed variations. Surface-attached microbial communities are known to provide important ecosystem services^2,4^ and, in tropical reefs, are considered important factors mediating larval settlement^11,41^.

Biofilms in early successional stages appear to be less specialized in the use of organic carbon sources than mature biofilms; indicating that the capacity to use a wide range of organic compounds might be advantageous for pioneering species^59^. Further, young biofilms are thought to be colonized by metabolic generalists that source dissolved organic carbon from the surrounding water column^60,61^. While our experiment did not capture the initial pioneering species (hours to days), indicator taxa of our early time points (2 weeks) included known copiotrophic bacteria, capable of utilizing a wide range of carbon compounds, including three OTUs of Bacteroidetes (Fig. 5). The marine Bacteroidetes are important decomposers and lauded for their ability to breakdown high molecular weight organic matter; two of these OTUs were further identified as Saprospiraceae, a family that is typically only found in surface attached communities in the marine environment, and includes species known to prey on microalgae^62^. Consistent with previous studies that captured these time frames, Alphaproteobacteria were also abundant in early biofilm communities^41^; and in our study were further identified as Rhizobiales, regarded as important mediators of biofilms formation^63^, and Caulobacterales, a well-established group for surface adhesion and monolayer formation^64,65^.

Over time, Gammaproteobacteria, Planctomycetes, and Acidobacteria increased in abundance supporting previous findings that indicate these groups differentiate biofilms from the free-living microbial community^41,66^. Important taxa enriched in later time points included the Alphaproteobacteria and Gammaproteobacteria families Rhodobacteraceae and Cellvibrionales both associated with polysaccharide decomposition. The Rhodobacteaceae are well-established late-successional colonizers of nutrient rich habitats^67^ while the recently established order Cellvibrionales^68^ includes both established polysaccharide degraders from soil habitats^69^ and a number of widespread marine Gammaproteobacterial clades (OM60/NOR5, SAR92) variously documented in diatom blooms associated with both upwelling^70,71^ and Southern Ocean iron enrichments^72^. These observations are consistent with well-established concepts of biofilms as communal digestive systems^73^, where resources from the surrounding environment are captured and interact with enzymes produced within the biofilm community to efficiently cycle nutrients^4,74^ and mature biofilms facilitate greater species diversity through habitat diversification and a wider array of resources^39^.

Resources provisioned from the surrounding benthic community, presumably dissolved organic matter^17,33,75^, were the strongest environmental influence on the structure and succession of marine biofilm communities. The bioavailability of DOC has been demonstrated to influence community composition in mature biofilms^7^ and ultimately affects the uptake and utilization of other nutrients^76^. DOC sourced from algal exudates are known to differ from that of corals in both quantity and composition^33,75^ and these differences result in a restructuring of the bacterioplankton community from highly diverse taxonomic assemblages to less diverse communities adept at quickly growing on labile carbon compounds^19,33^. Similarly, while bacterial evenness increased over the course of the experiment, biofilms cultured with sand and algae did not significantly increase in richness (Fig. 4). Further, OTUs indicative of biofilms cultured with algae were dominated by known copiotrophs (Fig. 5). In a sister study analysis of dissolved organic matter (DOM) from samples collected concurrently with our biofilm samples revealed that all benthic organisms in our study increased organic matter exudates in response to nutrient addition and corals exude more proteinaceous organic matter than other benthic organisms—specifically, the exudation and accumulation of tryptophan-like, tyrosine-like, and phenalalanine-like organic matter was enriched in coral treatments^17^. Tyrosine and tryptophan along with other small cyclic compounds have been shown to inhibit biofilm formation^77^ by inhibiting cellular communication^78,79^ and flagellar motility^80^. While reduction in biofilm formation and successional dynamics was not observed, it was clear from our study that coral and algal biofilms were distinct throughout the experiment and followed different successional trajectories. Furthermore, proteinaceous organic matter was always enriched in coral treatment relative to the influent seawater but decreased between the two-week and four-week time points^17^ and this reduction in organic matter coincides with the shift between early and late microbial communities, where coral biofilms differentiate from biofilms cultured with sand and algae. Interestingly, biofilm communities cultured in the presence of corals were enriched with several taxa known to be important in the breakdown of polycyclic hydrocarbons including the Rhodobacteraceae and Sphingomonadaceae families of Alphaproteobacteria, as well as the Gammaproteobacteria *Marinobacter*, suggesting that polycyclic hydrocarbons comprise a distinct pool of compounds released more by corals than by algae. Together our results provide clear evidence that biofilms also are strongly influenced by the composition of dissolved organic matter in coral reef systems.

The addition of inorganic nutrients increased the exudation of DOC by each of the benthic organisms in a companion study conducted coincident with this one in these same mesocosms^17^. While nutrient concentration was consistently a significant factor shaping biofilm communities, the nutrient effect on biofilms was relatively small and tended to primarily interact with other experimental parameters rather than serve as a strong primary control over biofilm composition. This emphasizes that organic matter quality, rather than quantity, is likely the dominant structuring force in biofilm composition. In our study, nutrients impacted both measures of diversity (richness and evenness) at 6 weeks. Because of the relatively weak role of nutrients in structuring communities we selected a singular time point and organismal treatment within, which we contextualized the impact of nutrient amendments: while evenness significantly increased through time in each of the organismal treatments, a significant increase in richness was observed only in biofilms cultured with corals at 6 weeks, in part due to interactions with nutrient enrichment. During week 6, nutrient additions decreased richness in biofilms cultured with algae but had no significant effect on evenness. For the same time period, nutrient additions had opposing effects on alpha diversity metrics in biofilms cultured with corals: richness increased with nutrients while evenness decreased. Furthermore, with increased nutrients, populations of bacteria typically associated with oligotrohic coral reefs decreased, including groups of Alphaproteobacteria and Cyanobacteria (e.g., Rhodobacteraceae and Thalassobius, and Rivularia and Synechococcus)^33,81^ and shifted toward populations of copiotrophic Flavobacteria.

In freshwater systems bacterial communities have been shown to preferentially utilize inorganic nitrogen sources in the presence of labile organic matter, however when concentrations of labile organic carbon are low, organic nitrogen from recalcitrant carbon sources is preferred as it might serve as both a carbon and nitrogen source for the bacterial community^76^. With evidence that coral exudates are enriched with DOM exhibiting fluorescence characteristics similar to aromatic amino acids^17^, we can hypothesize that at least some portion of the carbon compounds exuded by corals are relatively enriched in nitrogenous compounds. Functional genes directly related to the degradation of aromatic hydrocarbons have recently been linked with various metabolic pathways including those required for nitrogen fixation and sulfate metabolism^82^. It is interesting that OTUs from two groups of late-succession marine Bacteria established to metabolize polycyclic compounds, Sphingomonadaceae and Rhodobacteraceae^5,78,83^ respond differently to nutrient additions (Fig. 6); with the former increasing and the latter decreasing), potentially suggesting differences in nutrient requirements or capabilities between the two groups that imply shifts in the aromaticity of polycyclic DOM released by corals under nutrient enrichment. Such a shift would be consistent with our earlier observations of nutrient amendment altering the composition of coral exudates to increase humic-like components with potentially higher aromaticity^17^.

In coral reefs, anthropogenic stressors including overfishing and nutrient pollution have been implicated in phase shifts from coral dominated reefs toward those dominated by fleshy algae^28,84^. Previous research working to identify the underlying mechanisms contributing to these phase shifts have found both direct and indirect effects of algae on coral vitality^85,86^, and suggested that alterations in microbial community structure could create a feedback loop that maintains the shift from coral to algal dominance^19,34,87,88^. While this is likely only one contributing factor, variation in organic matter exudates produced by algae have been shown to be compositionally distinct from those of corals, resulting in a restructuring of the bacterioplankton community from highly diverse taxonomic assemblages to less diverse communities adept at quickly growing on labile carbon compounds^19,33^. In our study, biofilms cultured with algae were enriched in copiotrophic bacteria throughout the experiment (Fig. 5), including several types of Rhodobacterales and Flavobacterales, and nutrient additions correlated with decreased richness in mature algal biofilms (Fig. 4b), suggesting further bias toward a few dominant copiotrophs. Conversely, in biofilms cultured with corals, richness increased throughout the experiment and this trajectory was enhanced by the addition of nutrients. However, in mature biofilms cultured with corals, nutrient additions corresponded with a significant decrease in evenness (Fig. 4b) and increases in populations of Flavobacteriaceae (Fig. 6) suggesting that the addition of nutrients can derail some aspects of microbial diversity even in coral-dominated reefs by promoting the growth of copiotrophic organisms. Nutrient enrichment may be a key factor in initiating these phase shifts, and nutrient enrichment in reef waters increases exudate output from benthic community members and shifts bacterioplankton communities toward less diverse assemblages with increased virulence factors^19,33^. Nutrient enrichment is also implicated in the progression of coral disease^89^ and increased virulence factors of microbes inhabiting the surface mucus layer of corals^90^. Taken together, our results indicate that both benthic community structure and nutrients work orthogonally and interactively to influence the composition of coral reef biofilms, suggesting that ongoing microbialization of coral reefs is likely to alter biofilm structure and function.

Biofilms are known to host complex communities of microorganisms that work in concert to perform biogeochemical processes and ecosystem services. In this study, we demonstrate that marine biofilms differentiate from the planktonic community and exhibit successional trajectories distinct from their planktonic counterparts. Our results show that differences in organic matter produced by benthic organisms influence marine biofilms from early developmental stages and further differentiate these communities over time. This study further provides evidence that inorganic nutrient additions can shift biofilm communities either through the stimulation of primary producers, thereby reinforcing diverging microbial communities, or by shifting nutrient dependence away from biofilm derived sources, toward environmentally available inorganic sources. Our findings add to the growing evidence that chronic nutrient enrichment of reef ecosystems results in loss of diverse microbial assemblages. Finally, our work illustrates the structure of biofilm communities distinctly associated with coral and algal dominated reefs, paving the way for future understanding of how ongoing global phase shifts in coral ecosystems may impact key microbial processes crucial for reef resilience in a changing world.

## Methods

### Sample collection

Samples of coral, macroalgae, and carbonate sand were collected from the fringing reef around Moku o Lo’e (Coconut Island) adjacent to the Hawai’i Institute of Marine Biology (21.435°, −157.787°) on October 12–16, 2015. Collections were in accordance with local regulations; corals were collected under the State of Hawaiʻi Division of Aquatic Resources Special Activity Permit 2015–2017 to the Hawaiʻi Institute of Marine Biology. Corals were collected from the fringing reef on the southwest side of the island. Three individual colonies from the two dominant coral species in Kāneʻohe Bay, *Porites compressa*, and *Montipora capitata*, were harvested and fragmented to produce 36 coral nubbins, 12 from each of 3 colonies. Each nubbin was buoyant weighed and equally sized nubbins (*P. compressa* = 24.8 ± 5.23 g dry weight; *M. capitata* 21.9 ± 5.05 g dry weight) were mounted on polystyrene frames using epoxy. Each coral frame held six nubbins (three *P. compressa*, and three *M. capitata*); coral nubbins were allowed to acclimate for 10 days prior to the start of the experiment. Macroalgae (*Gracilaria salicornia*) and sand samples were collected from a low energy, sandy reef flat on the northern side of Coconut Island in less than 1 m depth, where *G. salicornia* is abundant and grows unattached to the substrate. Macroalgae samples were cleaned of visible invertebrates and epiphytes then wet weighed and split into 36 equal portions (11.0 ± 0.55 g wet weight) and contained in polyethylene mesh boxes. Samples of carbonate sand were collected using a 7.5 cm diameter core, placed in 36 petri dishes, then placed undisturbed in experiment aquaria. More detailed information on sample collection is available from Quinlan^17^ and Silbiger^32^.

### Experimental design

Experimental aquaria were set up in an outdoor mesocosm facility and consisted of 27 6 L, flow through, acid washed, polycarbonate aquaria which were divided between three, 1300 L, shaded incubation tanks (nine aquaria per tank) used to maintain constant temperature (Fig. 1 and Supplementary Fig. 1). Source water from Kāne’ohe Bay flows into the mesocosm facility first through a sand filter and 20 μm polyethylene cartridge filter before use in our experiment. The filtered seawater was subsequently pumped into nine nutrient mixing header aquaria (10 L) via multi-channel peristaltic pump; header mixing aquaria (three replicate aquaria of each of three nutrient treatments) were maintained at a 30-min residence time, cleaned weekly and variously housed 5–15 small (3 cm) coral fragments associated with a separate experiment. To create nutrient treatments in header aquaria, a bulk nutrient stock of potassium nitrate and potassium phosphate (3:1 molar *N*:*P*) was mixed at the beginning of the experiment and frozen in single use aliquots to maintain continuity throughout the experiment. Every other day, an aliquot of frozen nutrient stock was diluted to the appropriate concentration in seawater and administered to the header aquaria via peristaltic pump to mix. Treatments were maintained at three stable levels, including ambient (averaging 0.1 µM L^−1^ NO_3_^−^ and 0.06 µM L^−1^ PO_4_^3−^) and medium and high enrichments averaging 2.68 and 6.64 µmol L^−1^ NO_3_^−^, respectively^17,32^. Nutrient concentrations and *N*:*P* stoichiometry (2.46 ± 0.37 SD across all treatments) spanned natural inorganic nutrient conditions measured on reefs across the Hawaiian archipelago^91^ and background nutrient conditions during the experiment were consistent with baseline concentrations in Kāne’ohe Bay^92^. Each aquarium held one of the three benthic organisms (either four coral frames, four algal mesh boxes, or four petri dishes of sand) and received one of the three nutrient treatments (ambient, medium, or high nutrient addition) resulting in nine factorially-crossed treatments. Treatment aquaria were maintained at a 5 h residence time and mixed with a submersible water pump. Each set of nine treatment aquaria was established in one of three independent 1300 L flow-through incubator tanks to maintain thermal stability and was monitored over the course of 6 weeks. This resulted in a total of 27 aquaria comprising three independent replicates of each of the nine treatments. Each nutrient treatment level in a 1300 L tank was fed by an independent mixing header tank to ensure independence. To mitigate tank effects due to weather conditions and variations in light exposure; aquaria sets (blocks of nine aquaria) were rotated between, and individual aquaria were shuffled randomly within, the larger incubator tanks once per week over the course of the 6 week experiment. All plastics used in this experiment, including aquaria, slide racks, and tubing were acid-washed and soaked for at least 72 h in flowing seawater to remove plasticizers before starting the experiment.

### Biofilm culturing and sampling

Glass slides have been previously shown to provide suitable substrate for marine biofilms and are known to result in more consistent and reproducible communities compared with ceramic tiles^93^. Therefore, biofilms were cultured using glass slides suspended in the upper 10 cm of each experimental aquarium (Fig. 1). Slides were cleaned with alcohol and combusted to remove surface coatings and organic matter prior to the experiment. Slides were evenly spaced in polyoxymethylene slide racks typically used for microscopy staining, with ample space between each slide to allow for water to flow freely around each side of the glass slide. Racks were suspended using nylon line and polypropylene suction cups. Biofilm samples were destructively sampled by removing one glass slide from each aquarium rack at each sampling point in a manner intended to minimize altering the fluid dynamics surrounding the remaining slides. Sterile polyester tipped swabs (Puritan 25-806 1PD) were used to sample biofilms from each slide; a standardized swabbing technique was used to minimize variability between samples and time points: Each side of a slide was swiped ten times with a swab, turning the swab one quarter turn every five swipes. Slides were discarded after collection, and swab tips were placed in sterile tubes and frozen until DNA could be extracted. For analysis of planktonic bacteria, at each time point 40 mL of seawater was collected from each of three replicate aquaria within each organism by nutrient treatment using an acid washed, rubber-free polyethylene syringe, pooled into a single 120 mL sample and filtered through a 0.22 μm PES filter, resulting in nine DNA samples from planktonic organisms at each time point (27 planktonic samples total). Filters were frozen at −80 °C until DNA could be extracted. Genomic DNA from biofilm swabs and filters was extracted using the Epicenter MasterPure Complete DNA and RNA Purification Kit (MCD85201) using the protocol outlined by the manufacturer for DNA purification from plasmid or serum samples with the following modifications: samples were rotated throughout the lysis incubation and swabs or filters (depending on sample type) were aseptically removed following the addition of RNase and just before DNA precipitation steps. DNA was re-suspended in 50 μL TE buffer. Analysis of dissolved organic matter (DOM) was performed using the 0.22 μm PES filtrate collected synoptically with the samples described here. Findings from the DOM analysis are reported separately^17^.

### Bacterial SSU rDNA (16S) amplicon sequencing and analysis

The V3–V4 hypervariable regions of the 16S rRNA genes were amplified using primers 341F and 806R^94^, with unique paired end oligonucleotide sequence “barcodes” assigned to each sample as described in Kozich 2013. Polymerase Chain Reaction was performed in 25 μL volumes with 1 μL of DNA template or no-template control. Reaction conditions consisted of an initial denaturing step at 98 °C for 1 min followed by 30 cycles of denaturation at 98 °C for 15 s, annealing at 55 °C for 30 s, elongation at 72 °C for 30 s, with a final extension at 72 °C for 1 min. Equimolar amounts of amplicons from each sample were mixed and purified using the SequalPrep™ Normalization Plate following the manufacturerʻs protocol and submitted to the Hawaiʻi Institute of Marine Biology Evolutionary Genetics Core Facility for 600 cycle paired-end sequencing using the Illumina MiSeq V3 chemistry.

Raw sequence data was pre-processed into amplicon sequence variants (ASVs) at 100% nucleotide identity using the dada2 package in R version 4.0.3^95,96^. Reads were truncated at position 260/190 (forward/reverse) and were discarded if they contained one or more bases with quality scores less than 2, or more than 3 expected errors using the *filterAndTrim()* function. The *learnError()* and *dada()* functions were used with default parameters to denoise, and reads were merged using the *mergePairs()* function. Any pairs containing more than one mismatch or an overlap of fewer than 20 bases were discarded. Sequence alignment and annotation were performed in mothur v1.42.3^97^ using the SILVA.nr V132 SSU database^98^. Sequences with start or stop positions outside of the over-all 5th–95th percentile range were discarded. Potential chimera sequences were removed with *chimera.vsearch()*. Taxonomies were assigned using the *classify.seqs()* and *classify.otus()* functions. We identified, quantified, then removed from further analysis all mitochondrial or chloroplast OTUs, as well as sequences without at least a domain level classification. For subsequent statistical analysis, alpha-diversity, and beta-diversity we randomly subsampled at 3300 sequences per sample using the *sub.sample()* function. Samples containing fewer than 3300 sequences were discarded. We defined microbial operational taxonomic units (OTUs) as unique sequences (commonly referred to as amplicon sequence variants or ASVs) using dada2 and refined in R using the lulu package^99^. OTUs were merged if they co-occurred in every sample and one of the two ASVs had a lower abundance than the other in every sample. Finally, we discarded unreplicated OTUs (represented by two or less identical sequences across all samples). We further culled OTUs by discarding those with ten or fewer sequences, either across all samples or within a given sample. In all, our sample set consisted of 106 samples, including 79 biofilm samples and 27 water samples (Supplementary Table 1). Post-QC, gamma diversity for the dataset was 18,278 unique OTUs including 14,237 unique OTUs from biofilm samples compared with 4041 unique OTUs found in water.

### Statistical analysis

Multivariate analyses of time and treatment effects on biofilm and planktonic bacterial communities were performed on Bray–Curtis distance matrices constructed from OTU relative abundances using the *vegan* package in R^100^ including permutational analysis of variance (PERMANOVA) with the *adonis()* function and nonmetric multidimensional scaling with the *metamds()* function. Additional pairwise PERMANOVA tests were conducted for biofilm samples within each time point using the *pairwise.adonis* package^101^. For comparison, all multivariate statistical models were additionally run with weighted Unifrac distance matrices^102^ and yielded nearly identical results (Supplementary Table 2). When performing multiple tests, the false discovery rate was controlled by adjusting *p*-values according to Benjamini and Hochberg^103^. Linear univariate mixed-effect models were performed using the *lme4* and *lmerTest* packages in R^104,105^. Each experimental parameter: time point, benthic organism, and nutrient level, as well as factorial interaction terms, were included as fixed effects in the models, with holding tank and aquarium included as orthogonal random effects to account for environmental differences between experimental holding tanks (Fig. 1) and repeated measures, respectively. These models were used to determine the response of alpha diversity metrics (richness and evenness) as well as each bacterial population in biofilm samples. Prior to statistical analysis, relative abundances of bacterial taxa were angular transformed (arcsine of square root) to best approximate the gaussian distributional assumptions of the model and the false discovery rate was controlled by adjusting *p*-values according to Benjamini and Hochberg^103^. Bacterial populations found to have significant fixed effects for time or organism were then screened for associations to specific timepoints or organismal treatments using the *randomForest* function in R (randomForest package^106^) to assign discriminant scores (mean decrease in accuracy; MDA). For example, taxa with a significant fixed effect of time (FDR-adjusted *p* < 0.05) were evaluated for their predictive power (MDA score) in a random forest model for the three timepoints. Similarly, to identify taxa that were indicative of biofilms cultured with each benthic organism, we selected OTUs from the linear mixed model that were significantly influenced by benthic organism (*p* < 0.05) or time–organism interactions (*p* < 0.05). Because a positive MDA score indicates a variable performed better than a random permutation of variables when classifying each sample, taxa with positive MDA scores greater than two standard deviations from the mean MDA were interpreted as strongly associated with a treatment category and selected for visualization. Because multivariate nutrient effects manifested most clearly in the biofilms incubated with coral, mixed-effect models testing for nutrient effects on bacterial populations (those with with significant nutrient or nutrient interaction fixed effects *p* < 0.05) were restricted to coral-associated populations: we pre-screened taxa for association with specific biofilm organismal treatment categories, first using random forest to evaluate all biofilm taxa for strong organismal associations, then selecting taxa with local variable importance scores (MDA) that were higher in coral treatments compared with either sand or algae. Thus, any taxon interpreted as associated with a particular fixed effect category was screened by both linear models and random forest for a robust and conservative assignment.

### Reporting summary

Further information on research design is available in the Nature Research Reporting Summary linked to this article.

## Supplementary information

Supplementary Information

Reporting Summary

## Data Availability

The data for this project is available through the National Center for Biotechnology Information (NCBI) Sequence Read Archive (SRA) under BioProject number PRJNA760540.

## Code availability

Computer code used in this study is available upon request from the corresponding author.
