# Supplementary material for: Coral reef biofilm bacterial diversity and successional trajectories are structured by reef benthic organisms and shift under chronic nutrient enrichment
Source: NPJ Biofilms Microbiomes. 2021 Dec 1;7:84. doi: 10.1038/s41522-021-00252-1 (PMC8636626; doi:10.1038/s41522-021-00252-1)
Supplement: Supplementary file 1 — Supplementary Information [file 41522_2021_252_MOESM1_ESM.pdf]

Experiment photos:

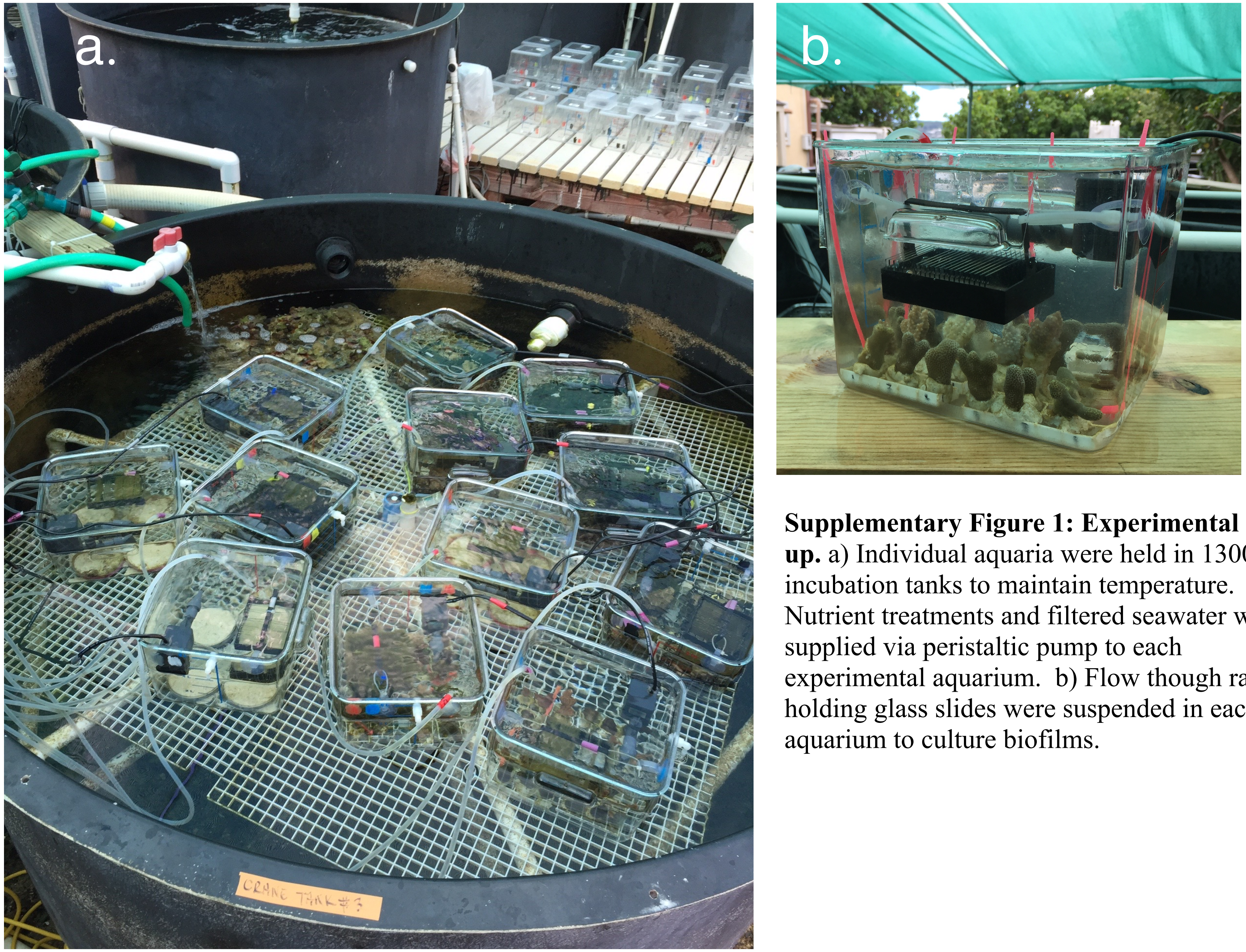

Supplementary Figure 1: Experimental set up. a) Individual aquaria were held in 1300L incubation tanks to maintain temperature. Nutrient treatments and filtered seawater were supplied via peristaltic pump to each experimental aquarium. b) Flow though racks holding glass slides were suspended in each aquarium to culture biofilms.

Supplementary Table 1

	Total Sequences	Total aligned sequences	Total unique aligned sequences	OTUs passing QC & >10 reads
Total	4,270,844	1,715,955	26,000	18,278
Biofilm N = 79	3,297,825	1,305,303	20,066	14,237
Planktonic N = 27	973,019	410,652	5,934	4,041

Supplementary Table 2

Comparison of Unifrac vs Bray Curtis distance matrices

Predictor	Whole Dataset				Water				Biofilm			
	Unifrac		Bray Curtis		Unifrac		Bray Curtis		Unifrac		Bray Curtis	
Results	R^2	P-Value	R^2	P-Value	R^2	P-Value	R^2	P-Value	R^2	P-Value	R^2	P-Value
	Results of Model 1				Results of Model 2				Results of Model 3			
Sample Type	0.284	0.001***	0.187	0.001***								
Time: Sample Type	0.100	0.001***	0.067	0.001***								
Time	0.126	0.001***	0.134	0.001***	0.651	0.001***	0.426	0.001***	0.242	0.001***	0.204	0.001***
Organism	0.056	0.001***	0.074	0.001***	0.064	0.001***	0.098	0.001***	0.115	0.001***	0.123	0.001***
Nutrient	0.030	0.001***	0.041	0.001***	0.067	0.001***	0.096	0.001***	0.061	0.001***	0.063	0.001***
Time:Org	0.051	0.001***	0.052	0.001***					0.106	0.001***	0.089	0.001***
Time:Nut	0.028	0.006**	0.039	0.001***					0.053	0.019*	0.060	0.07**
Org:Nut	0.024	0.001***	0.032	0.001***					0.047	0.001***	0.050	0.001***

Supplementary Figure 2: Biofilm dispersion between time points and for each time point between organism or nutrient treatment.

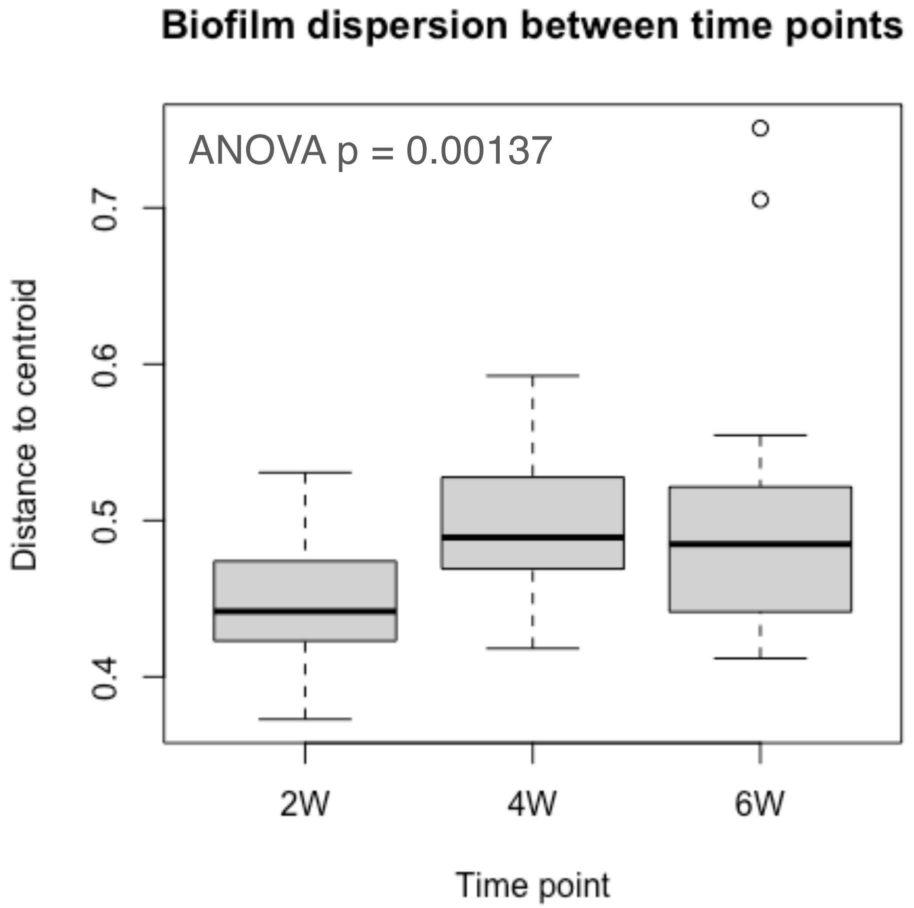

Results of Tukey HSD between time points

Time point	Adj. p-value
2 to 4 Week	0.00379
2 to 6 Week	0.00534
4 to 6 Week	0.993

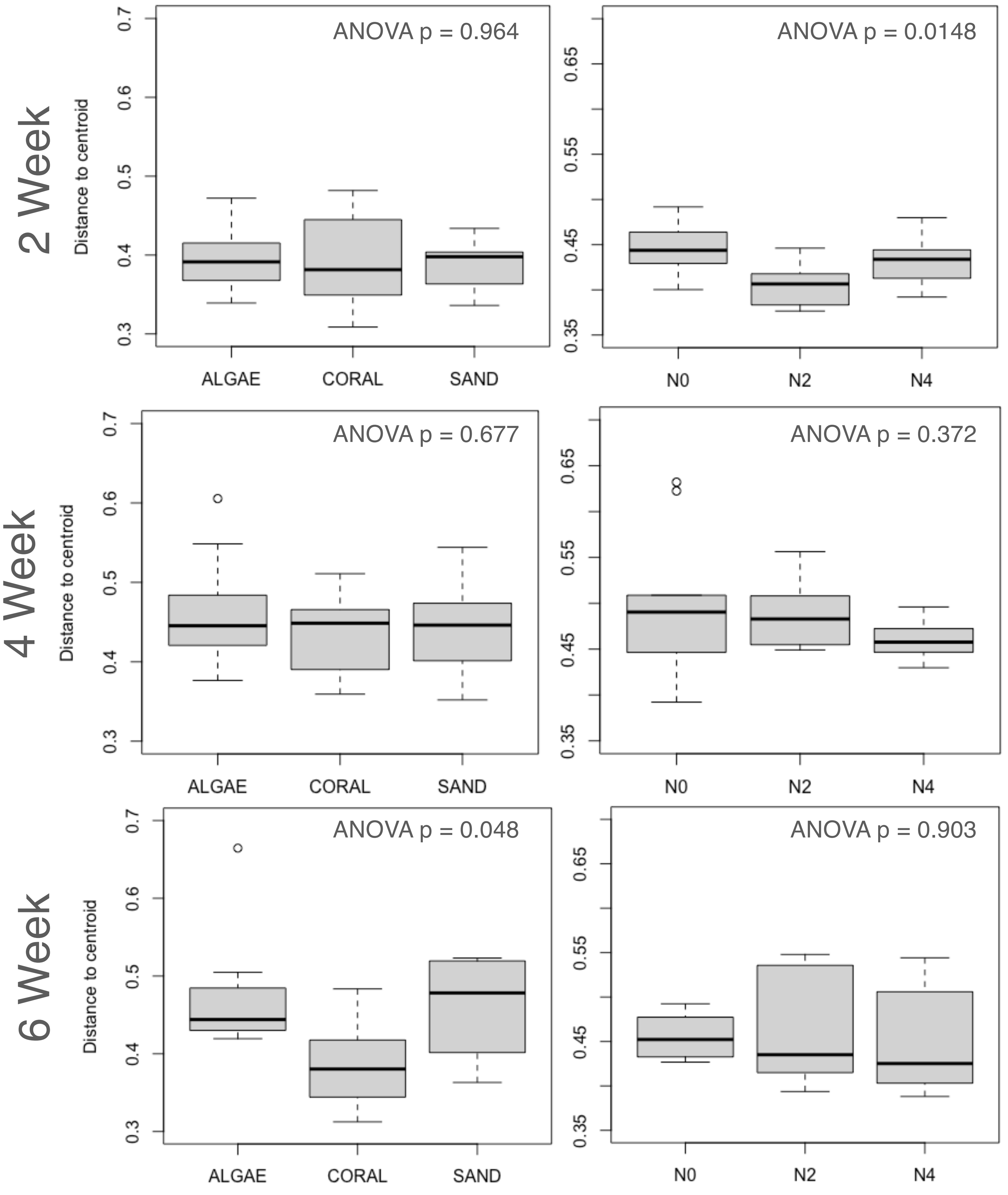

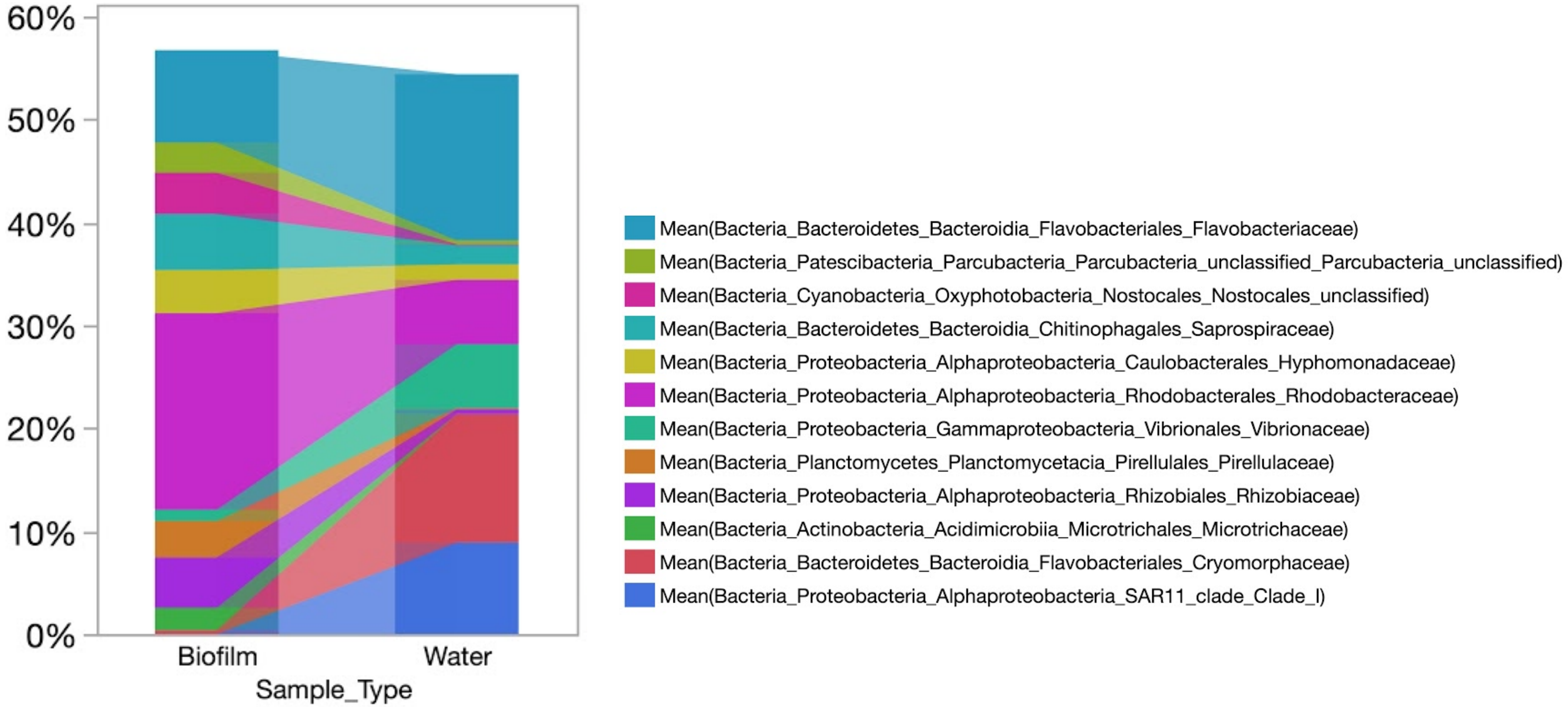

Supplementary Figure 3. Abundant families that differed significantly between biofilm and water samples. Means in each stacked bar are connected by lines to better visualize which taxa increase and which decrease in the context of the overall proportion of the community. Biofilms selected for The Alphaproteobacteria families Rhodobacteraceae, Rhizobiaceae, Hyphomonadaceae as well as the Parcubacteria, Nostocales, Microtrichaceae, Saprospiraceae and Pirellulaceae; Water samples were enriched in SAR11, Vibrionaceae and the Bacteroidetes families Flavobacteriaceae and Cryomorphaceae. Values are means of 79 biofilm and 27 water samples. Shown are the 12 families exceeding a mean of 2% in either sample type and differing significantly across all timepoints (one-way mixed-effect ANOVA with aquaria as a random intercept; FDR-adjusted $p < 0.05$); together they comprise more than half of the total sequence abundance of both sample types. Two additional families not shown, the Alphaproteobacteria Micavibrionaceae and the Gammaproteobacteria Cellvibrionaceae, comprised roughly 2% and 3%, respectively, of the sequences in both biofilm and water but did not differ significantly.

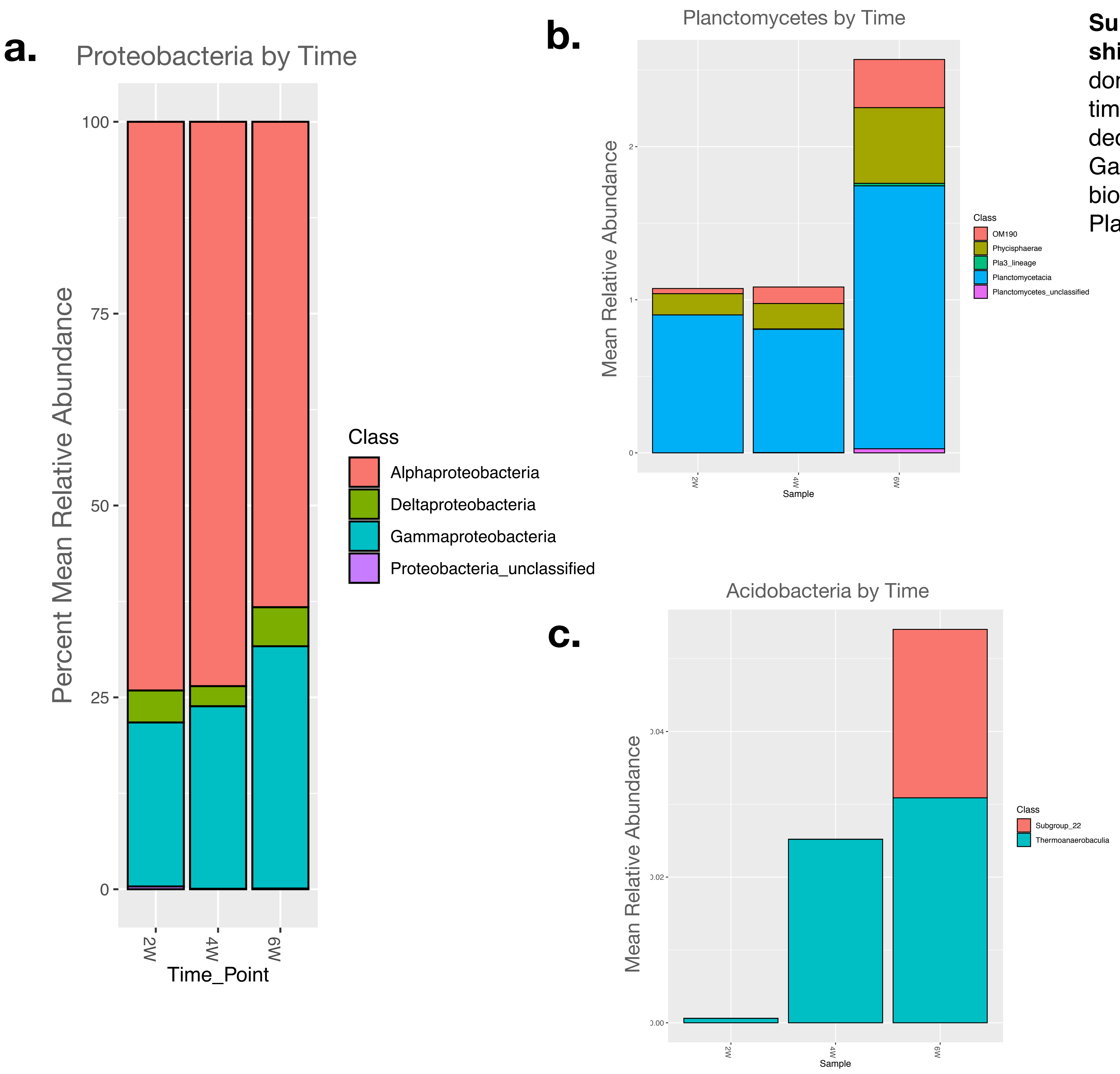

Supplementary Figure 4 Bacterial taxa shifts with time. Marine biofilms were dominated with Alphaproteobacteria at each time point. Over time, Alphaproteobacteria decrease, and an increase in Gammaproteobacteria is evident. Marine biofilms were further enriched with Planctomycetes and Acidobacteria with time.

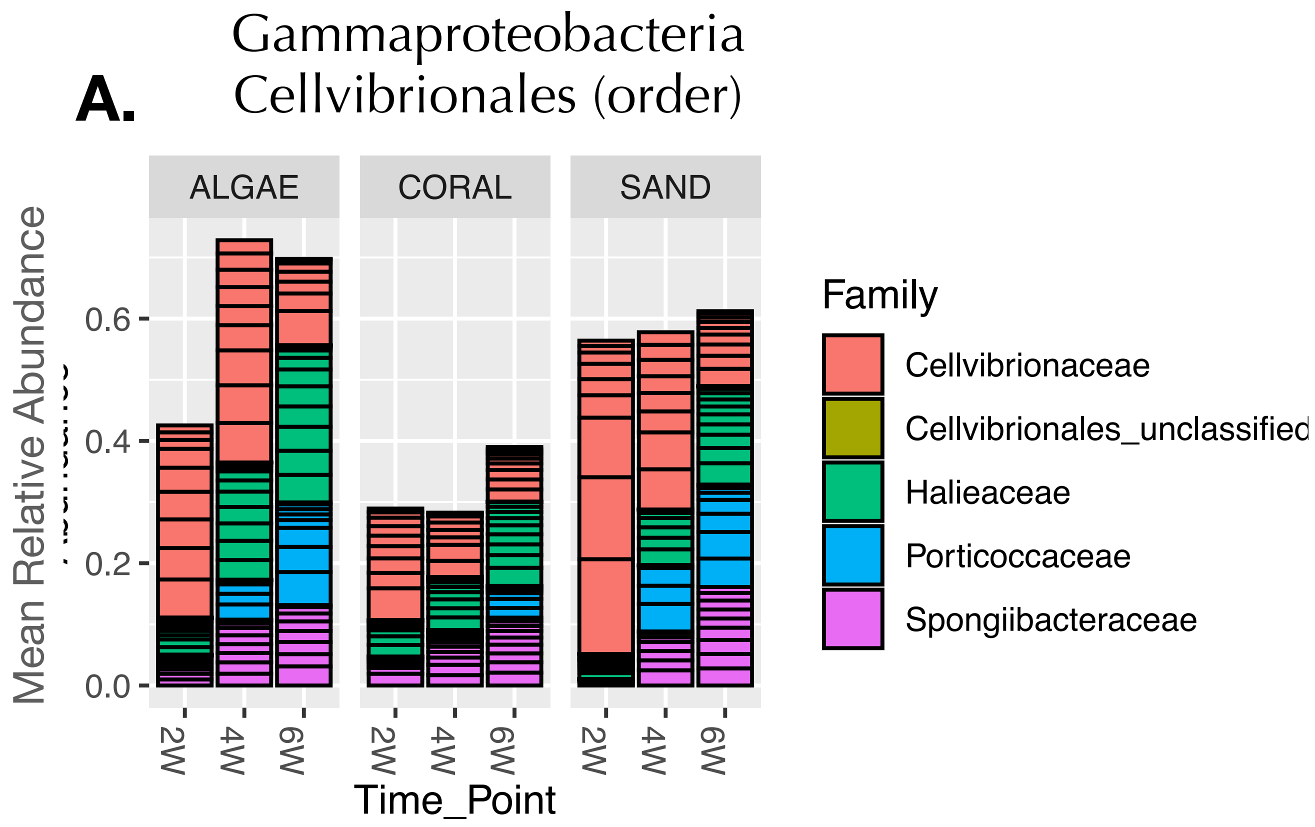

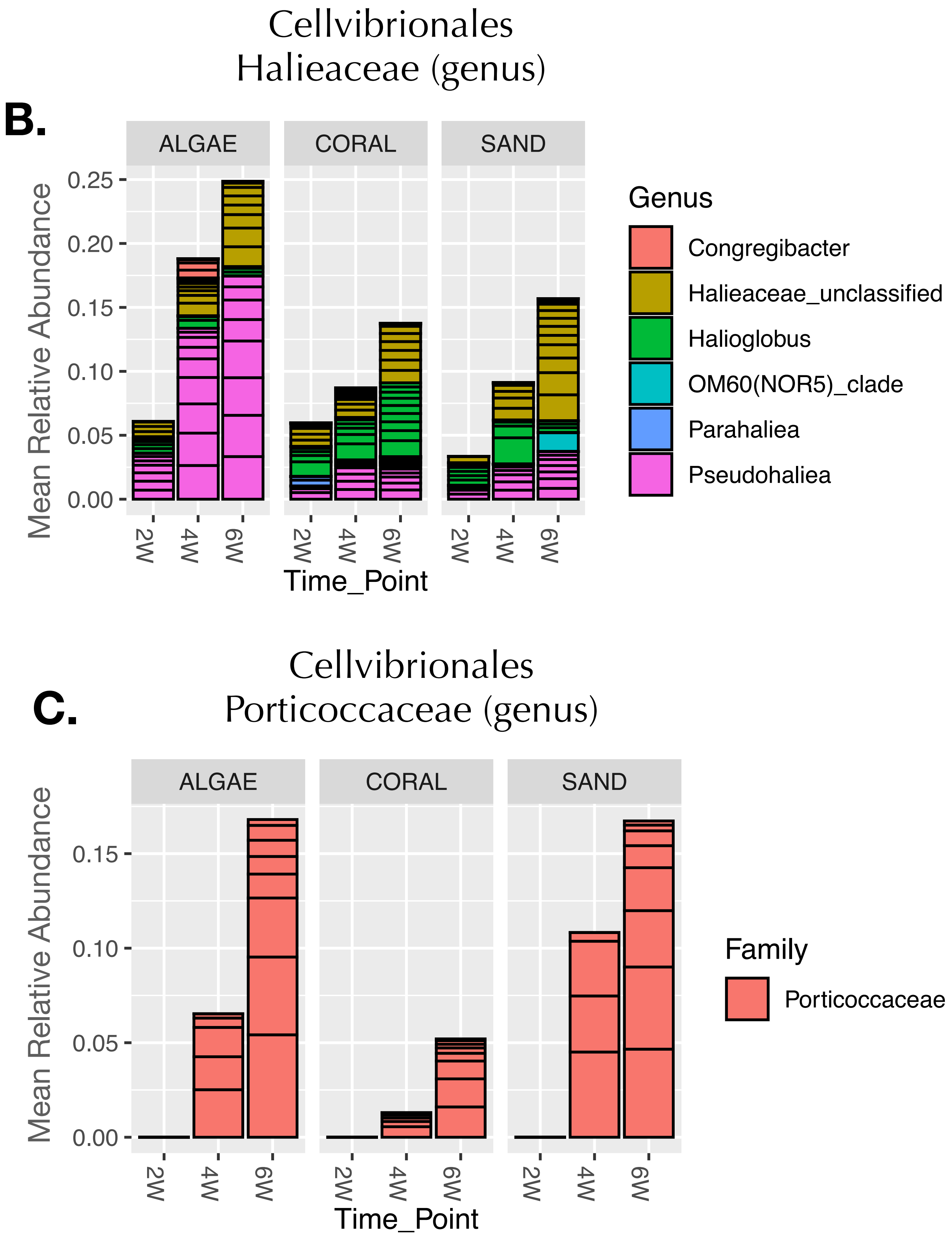

Supplementary Figure 5: Gammaproteobacteria increase in all biofilms as they mature. Within this class, Cellvibrionales were important indicators of biofilms cultured with sand and algae; and ASVs classified as Halieaceae and Porticoccaceae were enriched in algae and sand treatments.
