# Supplementary material for: Coral reef biofilm bacterial diversity and successional trajectories are structured by reef benthic organisms and shift under chronic nutrient enrichment
Source: NPJ Biofilms Microbiomes. 2021 Dec 1;7:84. doi: 10.1038/s41522-021-00252-1 (PMC8636626; doi:10.1038/s41522-021-00252-1)
Supplement: Supplementary file 2 — Reporting Summary [file 41522_2021_252_MOESM2_ESM.pdf]

nature portfolio

Corresponding author(s): Kristina Remple
Last updated by author(s): Oct 14, 2021

Reporting Summary

Nature Portfolio wishes to improve the reproducibility of the work that we publish. This form provides structure for consistency and transparency in reporting. For further information on Nature Portfolio policies, see our Editorial Policies and the Editorial Policy Checklist.

Statistics

For all statistical analyses, confirm that the following items are present in the figure legend, table legend, main text, or Methods section.

n/a	Confirmed	
☐	☒	The exact sample size (*n*) for each experimental group/condition, given as a discrete number and unit of measurement
☐	☒	A statement on whether measurements were taken from distinct samples or whether the same sample was measured repeatedly
☐	☒	The statistical test(s) used AND whether they are one- or two-sided<br>*Only common tests should be described solely by name; describe more complex techniques in the Methods section.*
☐	☒	A description of all covariates tested
☐	☒	A description of any assumptions or corrections, such as tests of normality and adjustment for multiple comparisons
☐	☒	A full description of the statistical parameters including central tendency (e.g. means) or other basic estimates (e.g. regression coefficient) AND variation (e.g. standard deviation) or associated estimates of uncertainty (e.g. confidence intervals)
☐	☒	For null hypothesis testing, the test statistic (e.g. *F*, *t*, *r*) with confidence intervals, effect sizes, degrees of freedom and *P* value noted<br>*Give P values as exact values whenever suitable.*
☒	☐	For Bayesian analysis, information on the choice of priors and Markov chain Monte Carlo settings
☐	☒	For hierarchical and complex designs, identification of the appropriate level for tests and full reporting of outcomes
☐	☒	Estimates of effect sizes (e.g. Cohen's *d*, Pearson's *r*), indicating how they were calculated

Our web collection on statistics for biologists contains articles on many of the points above.

Software and code

Policy information about availability of computer code

Data collection	Raw sequence data was pre-processed into amplicon sequence variants (ASVs) at 100% nucleotide identity using the dada2 package in R version 4.0.3
Data analysis	Statistical analyses were performed in R version 4.0.3 using the following packages: vegan pairwise.adonis, lmerTest, lme4, and randomForest

For manuscripts utilizing custom algorithms or software that are central to the research but not yet described in published literature, software must be made available to editors and reviewers. We strongly encourage code deposition in a community repository (e.g. GitHub). See the Nature Portfolio guidelines for submitting code & software for further information.

Data

Policy information about availability of data

All manuscripts must include a data availability statement. This statement should provide the following information, where applicable:

- Accession codes, unique identifiers, or web links for publicly available datasets
- A description of any restrictions on data availability
- For clinical datasets or third party data, please ensure that the statement adheres to our policy

The data for this project is available through the National Center for Biotechnology Information (NCBI) Sequence Read Archive (SRA) under BioProject number PRJNA760540.

Field-specific reporting

Please select the one below that is the best fit for your research. If you are not sure, read the appropriate sections before making your selection.

☐ Life sciences ☐ Behavioural & social sciences ☒ Ecological, evolutionary & environmental sciences

For a reference copy of the document with all sections, see nature.com/documents/nr-reporting-summary-flat.pdf

Ecological, evolutionary & environmental sciences study design

All studies must disclose on these points even when the disclosure is negative.

Study description	To contrast the roles of succession, benthic community structure, and nutrients in structuring marine biofilms, we surveyed bacteria communities in biofilms through a six-week succession in aquaria containing macroalgae, coral, or reef sand factorially crossed with three levels of continuous nutrient enrichment.
Research sample	Fragments of coral, Porites compressa and Montipora capitata; fleshy algae, Gracilaria salicornia; and carbonate sand were collected from a fringing reef on the island of Oahu in Hawaii. These are common benthic organisms and primary producers in coral reefs, and are representatives of competitive organisms in these environments.
Sampling strategy	Sample sizes were based on equal and comparable portions of each constituent benthic organism and constrained by aquarium size. Three full sets of experimental aquaria were established for statistical robustness.
Data collection	All authors contributed to collection of experimental parameter data. DNA sequence data was collected, processed, and curated by authors, Remple and Nelson,
Timing and spatial scale	Individual aquaria were set up and held benthic organisms starting 18, October 2015; organisms were allowed to acclimate for 1 week prior to the start of the experiment. The experiment started on 24, October 2015 when nutrients started flowing to each aquarium. Samples were collected every 2 weeks, and the experiment ended on 5, December 2015.
Data exclusions	From DNA sequences, OTUs identified as mitochondria and chloroplast, as well as any OTUs that were not classified to least at the domain level were removed from analyses. We then randomly subsampled at 3300 sequences per sample. Any samples with fewer than 3300 sequences were removed from subsequent analysis.
Reproducibility	Triplicate aquariums were set up for each combination of benthic organism and nutrient treatment.
Randomization	Three large incubation tanks were used for temperature regulation in our experiment. Each incubation tank held a set of experiment aquaria (as depicted in Figure 1 of the manuscript). Each week, coincident with aquarium cleaning, experiment aquaria were rotated through the 3 incubation tanks, and their placement was shuffled within each tank to minimize any effects of environmental variability.
Blinding	Because our experimental aquaria were randomized within and rotated between larger incubation tanks, we did not use blinding in this experiment.

Did the study involve field work? ☒ Yes ☐ No

Field work, collection and transport

Field conditions	Samples were collected from low energy, sandy reef flats on the northern side of Moku o Loʻe (Coconut Island).
Location	Samples were collected from the fringing reef on the southwest side of around Moku o Loʻe (Coconut Island) adjacent to the Hawaiʻi Institute of Marine Biology (21.435º, -157.787º).
Access & import/export	The experiment was conducted on island and did not need to be imported/exported. Samples were collected in accordance with all local regulations; corals were collected under the State of Hawaiʻi Division of Aquatic Resources Special Activity Permit 2015-17 to the Hawaiʻi Institute of Marine Biology.
Disturbance	*Describe any disturbance caused by the study and how it was minimized.*

Reporting for specific materials, systems and methods

We require information from authors about some types of materials, experimental systems and methods used in many studies. Here, indicate whether each material, system or method listed is relevant to your study. If you are not sure if a list item applies to your research, read the appropriate section before selecting a response.

Materials & experimental systems

n/a	Involved in the study
☒	☐ Antibodies
☒	☐ Eukaryotic cell lines
☒	☐ Palaeontology and archaeology
☐	☒ Animals and other organisms
☒	☐ Human research participants
☒	☐ Clinical data
☒	☐ Dual use research of concern

Methods

n/a	Involved in the study
☒	☐ ChIP-seq
☒	☐ Flow cytometry
☒	☐ MRI-based neuroimaging

Animals and other organisms

Policy information about studies involving animals; ARRIVE guidelines recommended for reporting animal research

Laboratory animals	*For laboratory animals, report species, strain, sex and age OR state that the study did not involve laboratory animals.*
Wild animals	*Provide details on animals observed in or captured in the field; report species, sex and age where possible. Describe how animals were caught and transported and what happened to captive animals after the study (if killed, explain why and describe method; if released, say where and when) OR state that the study did not involve wild animals.*
Field-collected samples	Samples were collected in accordance with all local regulations; corals were collected under the State of Hawaiʻi Division of Aquatic Resources Special Activity Permit 2015-17 to the Hawaiʻi Institute of Marine Biology.
Ethics oversight	Hawaii Department of Land and Natural Resources

Note that full information on the approval of the study protocol must also be provided in the manuscript.
